# Exploring the mechanism of BK polyomavirus-associated nephropathy through consensus gene network approach

**DOI:** 10.1371/journal.pone.0282534

**Published:** 2023-06-15

**Authors:** Noriaki Sato, Keita P. Mori, Kaoru Sakai, Hitomi Miyata, Shinya Yamamoto, Takashi Kobayashi, Hironori Haga, Motoko Yanagita, Yasushi Okuno

**Affiliations:** 1 Department of Biomedical Data Intelligence, Graduate School of Medicine, Kyoto University, Kyoto, Japan; 2 Department of Nephrology, Graduate School of Medicine, Kyoto University, Kyoto, Japan; 3 TMK Project, Medical Innovation Center, Graduate School of Medicine, Kyoto University, Kyoto, Japan; 4 Department of Nephrology and Dialysis, Medical Research Institute Kitano Hospital, PIIF Tazuke-Kofukai, Osaka, Japan; 5 Department of Urology, Graduate School of Medicine, Kyoto University, Kyoto, Japan; 6 Department of Diagnostic Pathology, Graduate School of Medicine, Kyoto University, Kyoto, Japan; 7 Institute for the Advanced Study of Human Biology (ASHBi), Kyoto University, Kyoto, Japan; Indiana University Purdue University at Indianapolis, UNITED STATES

## Abstract

BK polyomavirus-associated nephropathy occurs in kidney transplant recipients under immunosuppressive treatment. BK polyomavirus is implicated in cancer development and invasion, and case reports of renal cell carcinoma and urothelial carcinoma possibly associated with BK polyomavirus has been reported. Further, it has been suggested that the immune responses of KT-related diseases could play a role in the pathogenesis and progression of renal cell carcinoma. Thus, we thought to examine the relationship between BK polyomavirus-associated nephropathy and renal cell carcinoma in terms of gene expression. To identify the common and specific immune responses involved in kidney transplantation-related diseases with a specific focus on BK polyomavirus-associated nephropathy, we performed consensus weighted gene co-expression network analysis on gene profile datasets of renal biopsy samples from different institutions. After the identification of gene modules and validation of the obtained network by immunohistochemistry of the marker across kidney transplantation-related diseases, the relationship between prognosis of renal cell carcinoma and modules was assessed. We included the data from 248 patients and identified the 14 gene clusters across the datasets. We revealed that one cluster related to the translation regulating process and DNA damage response was specifically upregulated in BK polyomavirus-associated nephropathy. There was a significant association between the expression value of hub genes of the identified cluster including those related to cGAS-STING pathway and DNA damage response, and the prognosis of renal cell carcinoma. The study suggested the potential link between kidney transplantation-related diseases, especially specific transcriptomic signature of BK polyomavirus associated nephropathy and renal cell carcinoma.

## Introduction

BK polyomavirus (BKPyV) is a double-stranded DNA virus that was first isolated from a kidney transplant (KT) recipient’s urine sample. BK polyomavirus nephropathy (BKPyVAN) occurs in 8% of KT recipients [1], and one study reported that about 50% of BKPyVAN patients lost their allografts six months post-diagnosis on average [2]. BKPyVAN is treated by reducing immunosuppression, and definitive treatments are being discussed [3, 4]. Recent studies reported that BKPyV successfully replicates themselves by inducing DNA damage response (DDR) when the genome of the infected cell is damaged [5, 6]. Investigating the molecular backgrounds behind BKPyV infection is crucial for understanding the pathogenesis in detail.

Transcriptomic studies have been performed for KT-related diseases including BKPyVAN and T-cell mediated rejection (TCMR), which is one of the major problems after KT. One recent study concluded that it is not feasible to distinguish BKPyVAN and concurrent TCMR using microarray datasets, highlighting that two conditions have similar immune responses and the importance of comparing these conditions by taking the similarity and dissimilarity into account [7]. However, few studies have examined transcriptomic differences of these conditions across multiple datasets.

Further, it has been reported that renal cell carcinoma (RCC) is an immunogenic tumor, and the immune responses of these KT-related diseases could play a critical role in its pathogenesis and progression [8, 9]. The BKPyV infection has been reported to be related to cancer through several mechanisms including viral integration and sustained proliferative signaling [10]. However, whether there is a relationship between the identified transcriptomic signature of KT-related diseases, especially BKPyVAN, and RCC in terms of the prognosis of the patients remained unclear.

In this study, to assess the relationship between KT-related diseases and RCC, we first performed the consensus weighted gene co-expression network analysis (WGCNA) to elucidate the specific and common features of KT-related clinical conditions across different datasets and institutions, with a specific focus on BKPyVAN [11]. After the validation by immunohistochemistry (IHC), we assessed the relationship between identified clusters and RCC prognosis, thus exploring the potential link between KT-related diseases and RCC.

## Materials and methods

### Weighted gene co-expression network analysis

We first searched for molecular studies investigating BKPyVAN and summarized the result. Among them, we gathered and recategorized publicly available microarray and RNA-seq datasets related to BKPyVAN, and the details are summarized in S1 Text. We performed WGCNA to construct gene co-expression networks to identify consensus modules and hub genes playing important roles in KT-related diseases using microarray datasets. The detail of the analysis was summarized in S2 Text. Based on the calculated modules, we performed linear regression analysis with the module eigengenes (MEs) as the outcome variable, and disease status as the predictor variable, to identify which eigengene differed across the disease status for each dataset. The relationship between ME is depicted by the R library pvclust [12].

### The annotation of the modules and identifying the hub genes

Statistically significant modules were annotated using reactome using over-representation analysis by ReactomePA [13, 14]. The hub genes of the specific consensus modules were defined by kME (eigengene-based connectivity), which assessed intramodular connectivity, calculated by the function *consensusKME*. We defined those genes with the consensus kME values of 0.75 or above as hub genes in the module.

### The immunohistochemistry staining in renal biopsy samples

To validate the inferred network, we performed the IHC of a phosphorylated form of H2A histone family member X (γH2AX), which is one of the components of histone H2A. After DNA double-stranded breaks, H2AX is phosphorylated specifically at serine 139 and referred to as γH2AX [15]. We specifically examined γH2AX among the identified hub genes as it has been broadly used as a sensitive molecular marker to detect DNA damage and repair [16]. We performed IHC of γH2AX on paraffin-embedded needle renal biopsy specimens from two, one, and two patients with pathologists-diagnosed BKPyVAN, BKPyVAN with the suspected concurrent acute rejection (AR), and AR respectively. Additionally, as the negative and positive control, normal biopsy specimens obtained from the protocol biopsy of kidney transplantation, and a specimen of RCC were stained. For BKPyVAN specimens, SV40 staining was additionally performed on the serial section. IHC was performed on 4 μm deparaffinized tissue slices after antigen retrieval in the citrate buffer (pH 6.8) of 120°C for 15 minutes. The primary antibody used was γH2AX (Cell Signaling Technology, #9718) and SV40 (Sigma-Aldrich, PAb416). For indirect staining, Histofine Simple Stain MAX PO (Nichirei Biochemicals, Japan) and DAB Substrate Kit (Vector Labs, CA) were used. The slides were examined by BZ-X710 microscopy (Keyence, Japan). The study protocol was approved by the Ethics Committee on Human Research of the Graduate School of Medicine, Kyoto University (No. G0562 and R0254), and the study adhered to the Declaration of Helsinki. The written informed consent was obtained from the patients of KT biopsy samples. For RCC samples, written consent has been obtained for the study protocol No. R0097, and we provide opt-out information on the homepage of our department for research use of the study protocol No. R0254.

### Validation in RNA-seq dataset

We performed module preservation analysis using RNA-seq data investigating transplant kidney biopsies, and defining housekeeping genes for KT-related diseases [17, 18]. The dataset contains RNA-seq data of 30 kidney biopsies of 5 clinical conditions. We used DESeq-normalized count data as input. The modules discovered in consensus network analysis in the microarray network were tested for its preservation in RNA-seq dataset by comparing each microarray dataset and RNA-seq dataset. Additionally, module eigengene of the specific module was calculated in RNA-seq dataset, and compared between clinical conditions.

### The survival analysis

The log2 transformed, RSEM normalized transcript data and metadata of The Cancer Genome Atlas Kidney Renal Clear Cell Carcinoma was downloaded from UCSC Xena browser [19]. Kaplan-Meier plots were drawn to visualize the overall survival across the group divided by the first principal component of expression values of the gene signatures. The Cox proportional hazard models were deployed to derive hazard ratios for an association between gene signatures and overall survival. The genes with top-5 kME were used as the signatures for each module. Age, gender, tumor grade, and stratified pathologic stage were included as covariates. The pathologic stage and tumor grade are known to be prognostic factors of RCC [20]. The model fitting including the calculation of hazard ratio (HR) and its 95% confidence interval (CI), as well as the visualization, were performed by the R package survival and survminer [21, 22]. As reported in the literature [23], we performed the comparison with random gene signatures of the same number for the significant modules to assess the uniqueness of the signature on assessing the prognosis, using the function from SigCheck [24].

### Statistical analysis

All statistical tests were two-sided, and p-values, or p-values correctedby the Bonferroni procedure where appropriate, of less than 0.05 were considered statistically significant. The plots were generated using R libraries firatheme, ComplexHeatmap, and ggplot2 [25–27]. The network construction was performed by R library WGCNA and igraph [11, 28]. The visualization and the network export were performed by R package ggraph and Cytoscape [29, 30].

### Data availability

The microarray and RNA-seq datasets used in the study are available on the NCBI GEO database under the accession numbers GSE47199, GSE72925, GSE75693, and GSE120495 [31]. The sub-networks of the investigated modules are deposited in The Network Data Exchange and can be explored for the specific gene of interest. (https://www.ndexbio.org/#/networkset/9be9f058-4f49-11ed-ae36-0ac135e8bacf) [32].

## Results

### The weighted gene co-expression network analysis

**Fig 1:**
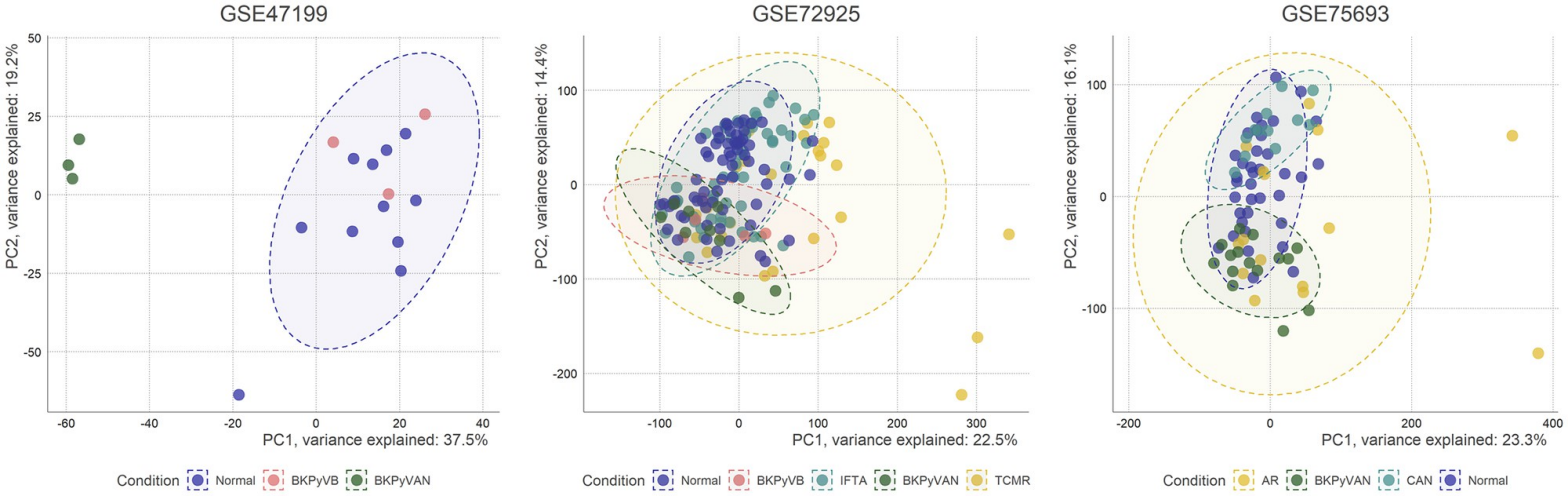
The results of the principal component analysis. The results of the principal component analysis of the samples for each dataset are presented. The x and y-axis represent the principal component one and two respectively. The color indicates the disease category, and the 95% confidence ellipses are drawn for each category. IFTA, interstitial fibrosis and tubular atrophy; Normal, normal biopsy; BKPyVAN, BK polyomavirus-associated nephropathy; BKPyVB, normal biopsy findings with BKPyV DNAemia; AR, acute rejection; TCMR, T-cell mediated rejection; CAN, chronic allograft nephropathy.

The molecular studies investigating BKPyVAN are summarized in Table 1. For consensus network analysis, we included the data of 17084 gene expression profiles of 154 patients in GSE72925, 77 patients in GSE75693, and 17 patients in GSE47199, after the preprocessing and filtering. The results of the principal component analysis for each dataset before merging the datasets were shown in Fig 1. For WGCNA, the relationship between soft threshold, scale-free topology fit index, and mean connectivity for each dataset are presented in S1 Fig. We detected 14 consensus modules in total by the power of 12. The dendrogram which shows the hierarchical clustering result of MEs for each dataset is presented in S2 Fig.

**Table 1:** The description of molecular studies analyzing clinically diagnosed BK polyomavirus nephropathy.

Authors	Number and type of samples	Background of kidney biopsy samples	Availability of dataset	Reference
Wang *et al*.	30 kidney allograft biopsies	5 ATI, BKVN, IFTA, ISN, STA, and TCMR	GSE120495	[17]
Adam *et al*.	110 kidney biopsies	BKVN and TCMR	Not available	[7]
Halloran *et al*.	102 kidney biopsies	50 BKPyVAN and 52 controls	Not available	[33]
Mannon *et al*.	42 kidney biopsies	15 normal kidney, 10 BKPyVAN, and 17 AR	Not available	[34]
Lubetzky *et al*.	17 kidney biopsies and 40 blood samples	3 BKVN, 3 BK viremia, and 13 stable graft function	GSE47199	[35]
Sigdel *et al*.	168 kidney biopsies	10 BKPyVAN, 26 TCMR, 59 IFTA, and 73 stable grafts	GSE72925	[36]
Sigdel *et al*.	79 kidney biopsies (compared with urine proteomics)	30 Standard, 15 AR, 15 BKPyVAN, 12 CAN, and 7 no CAN	GSE75693	[37]

AR, acute rejection; ATI, acute tubular injury; BKPyVAN, BK polyomavirus-associated nephropathy; BKVN, BK polyomavirus-associated nephropathy; CAN, chronic allograft nephropathy; IFTA, interstitial fibrosis and tubular atrophy; ISN, interstitial nephritis; STA, Standard; TCMR, T-cell mediated rejection.

### The relationship between module eigengenes and clinical conditions

**Fig 2:**
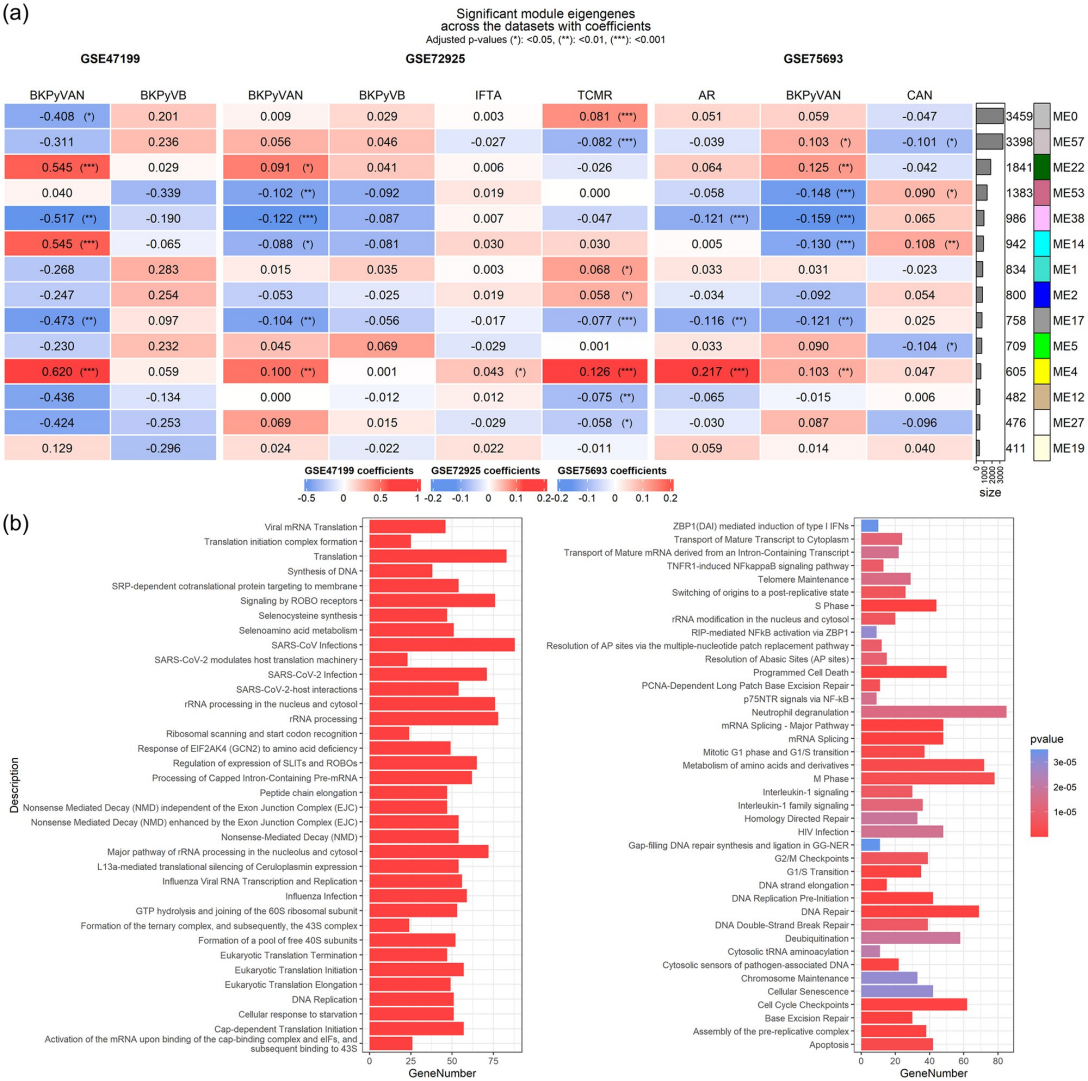
The relationship between module eigengenes and clinical conditions. (a) The relationship between clinical conditions and the module eigengenes is depicted for each dataset. The value and color of the cell of the heatmap represent coefficients of the linear models, and the asterisk mark indicates significance. Note that the range of the color value is specific to each dataset. The modules are sorted according to the number of contained genes. The color column represents the module color assigned by WGCNA. (b) The subset of significantly enriched pathways in specifically upregulated module in BK polyomavirus-associated nephropathy. The x-axis represents the number of genes that belong to the pathway. The color represents the values of the raw p-value.

We performed the linear regression analysis to assess the relationship between ME and clinical conditions. The overall result is shown in the heatmap depicting coefficients and statistical significance, along with the number of genes in the modules (Fig 2a).

We divided the significant modules into two categories. One category contained those modules that significantly differed in the same direction in AR or TCMR and BKPyVAN category in all datasets (common group), and the other contained significantly differed in the same direction only in BKPyVAN category in all datasets (specific group). The upregulated common group included ME4 (size 605), and interstitial fibrosis and tubular atrophy had also upregulated ME4. The ME17 (size 758) was downregulated in both conditions. The specific group included only ME22 (size 1841). We included these three modules for the downstream analysis.

### The module annotation

Three modules were first annotated by reactome using ReactomePA. Overall, 35, 3, and 76 reactome pathways were annotated for modules 4, 17, and 22 respectively. Module 22, which only belonged to the specific group, included the pathway of translation regulating process, cell cycle regulation, DNA replication, and DDR. The bar plot depicting the number of genes in the significant pathways in Module 22 is shown in Fig 2b.

Module 4, which belonged to the common group and upregulated, contained genes that were related to interferon signaling, T-cell receptor signaling, chemokine receptors bind chemokines, and the other ontologies like interleukin-family signaling. Module 17 was annotated for pathways like metabolism of vitamins and cofactors. All the statistical results are presented in S1 Table.

### The hub genes of each module

We identified the hub genes of the modules by the threshold of kME. Module 22 contained 76 hub genes, and Module 4 and 17 contained 27 and 39 respectively. We listed all the hub genes identified in S2 Table.

**Fig 3:**
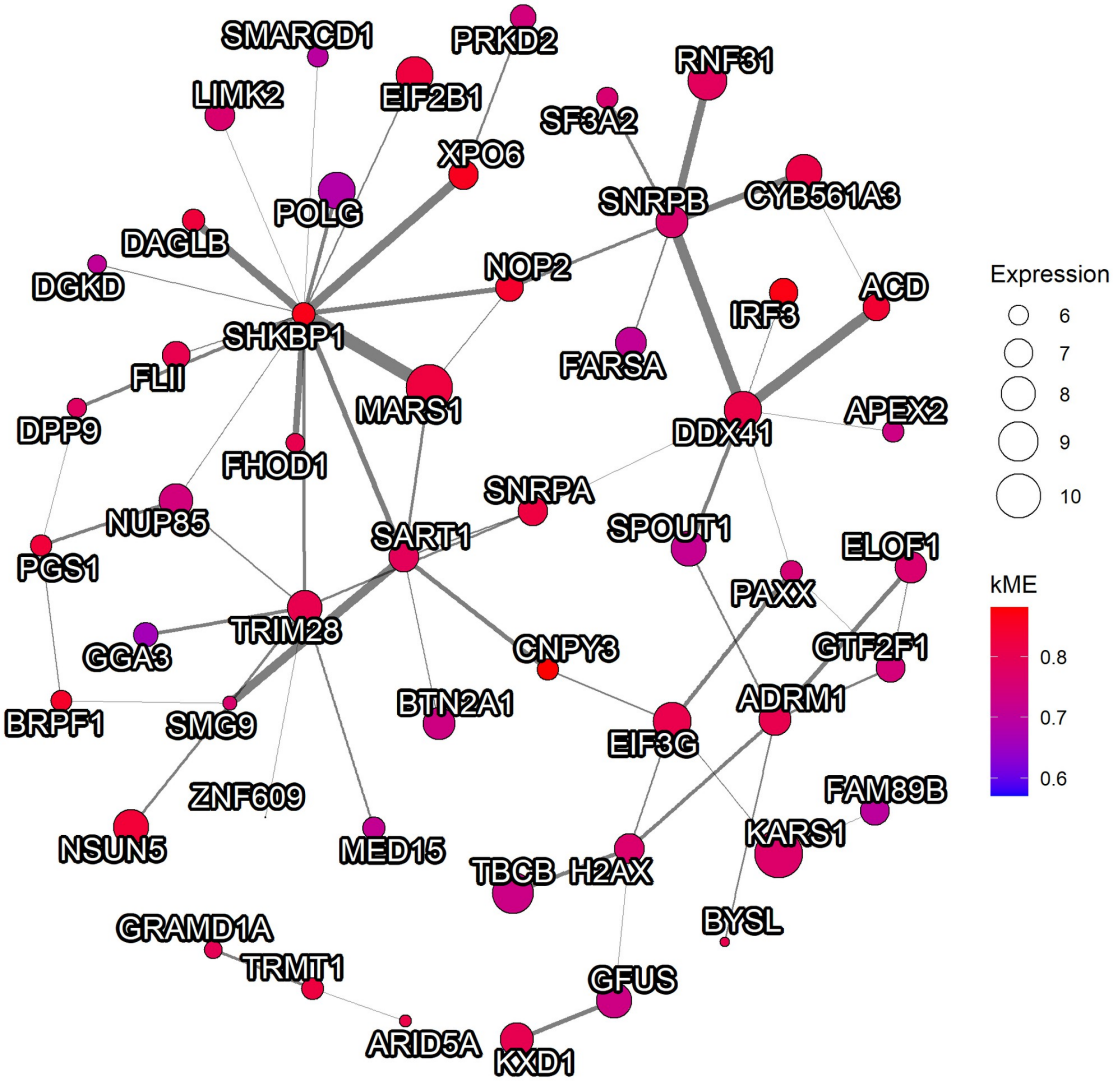
The visualization of sub-network of the module related to BKPyVAN. The sub-network of the top-5 scored hub gene and genes with the order of one from the gene within the corresponding module are visualized. The edges with the weight below the 99.9^th^ percentile and the nodes with zero degree were discarded beforehand. Additionally, only the edges with the weight above the 90^th^ percentile are included in the visualization. The color represents the consensus kME value. The edge width represents the strength of the connection between nodes. The node size represents the average normalized expression values of BK polyomavirus-associated nephropathy patients in GSE72925.

The hub genes of Module 22 contained those involved in the DDR pathway: DNA sensor DEAD-Box Helicase 41 (DDX41), H2AX, minichromosome maintenance complex components 5 and 7, poly-nucleotide kinase phosphatase (PNKP), and PAXX. Eukaryotic initiation factors, as well as the other genes involved in transcription regulation including elongation factor 1 are also included. We visualized the sub-network centered to the top-5 scored hub gene in module 22 (Fig 3).

The hub genes of module 4 included Lymphocyte Cytosolic Protein 2, Rac Family Small GTPase 2 and CD52. The hub genes of module 17 included eIF4E binding protein 2, solute carrier families of SLC22A5, and SLC25A44.

As the genes of Module 22 were annotated to DDR-related reactomes and contained DDR-related genes, we obtained the list of DDR-related genes [38], listed the subpathways of DDR for the genes, and plotted the proportion of DDR-related genes in the modules in S3 Table and S3 Fig. The result indicated that the genes involved in base excision repair and ribonucleotide excision repair were mainly in Module 22.

### Immunohistochemistry of renal biopsy samples

To examine the validity of the resulting network, we performed IHC of γH2AX, one of the identified hub genes, for the renal biopsy specimens obtained from patients with BKPyVAN, BKPyVAN with the suspected concurrent AR, AR, and normal findings, as well as RCC, which was used as a positive control. The demographic information and the detailed pathological diagnosis of the patients are shown in S4 Table.

For the BKPyVAN patient, most γH2AX-positive tubular cells were observed in the vicinity of SV40-positive tubular cells (S4a–S4e Fig). There was no γH2AX signal found where the SV40 signal was negative. On the patient with BKPyVAN with the suspected concurrent AR, a few tubular γH2AX signals were detected, however, SV40 was not detected in this specimen (S5a and S5b Fig). Unexpectedly, the sections cut out this time were negative for SV40 regardless of positive at the time of the original biopsy in this patient. We speculated loss of immunogenicity of SV40 or low specificity of γH2AX stains in long-term storage samples (S5c Fig).

On two AR patients, one patient got a strong γH2AX signal on the infiltrating cells (S4f Fig), however, only faint γH2AX signals in interstitial areas are observed with another patient with AR (S4g Fig). No evident signal was detected in infiltrating cells of the interstitial areas on the BKPyVAN patient. The specimen of RCC had a focal region of strong signal (positive control) and the control specimen (3 months protocol biopsy without any abnormality) showed faint signals (negative control) (S4h and S4i Fig).

### Validation in RNA-seq dataset

We next performed module preservation analysis comparing between each microarray dataset and RNA-seq dataset. The results are summarized in S6A Fig. The results indicate that the BKPyVAN-specific module is moderately to highly preserved in RNA-seq dataset. As we confirmed module preservation in RNA-seq dataset, we next calculated the consensus module eigengenes in RNA-seq dataset. We compared the module eigengene of module 22 across five disease conditions (S6B Fig), and found that BKPyVAN exhibits the highest mean value, while TCMR exhibits lowest. This result is in concordant with the result that corresponding module is upregulated in BKPyVAN, and not in TCMR, suggesting this module is pertinent to BKPyVAN across multiple datasets including microarray and RNA-seq.

### Survival analysis of renal cell carcinoma

**Fig 4:**
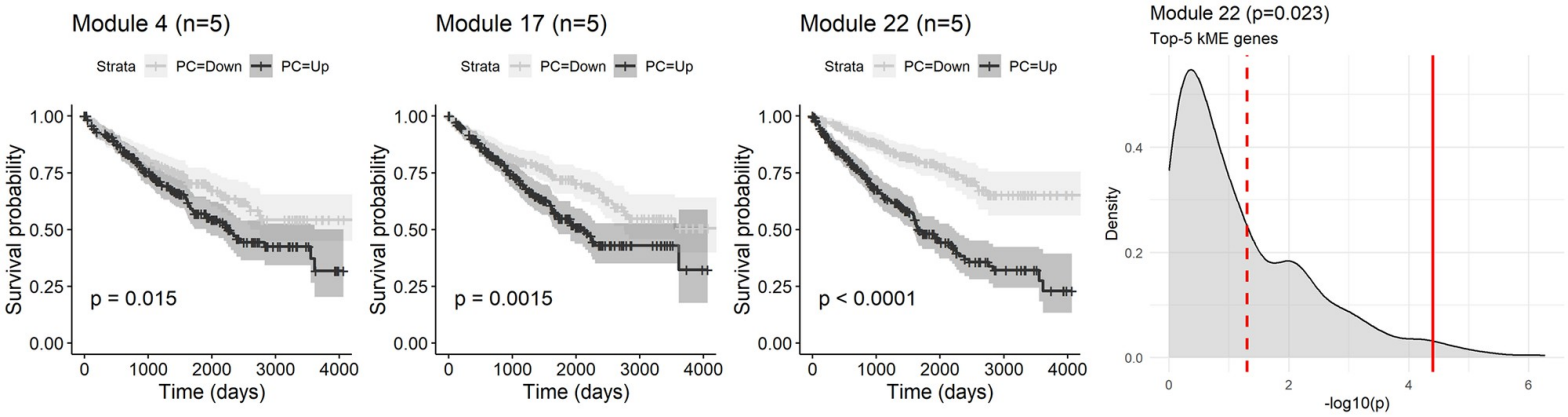
The summary of the survival analysis of the renal cell carcinoma. The Kaplan-Meier plots of the overall survival and the representative expression value of each significant module using genes with the top-5 kME values. The p-values of log rank test are shown. The density plot represents the distribution of p-values calculated by the same method using randomly selected genes with the same number. The solid red line indicates the p-value of the gene signature, and the dashed line indicates p-value of 0.05.

Finally, we performed the survival analysis of RCC using the expression values of identified three modules. The tumor grade of “GX” was excluded, and “G1” was merged into “G2” category as the number of samples was small. We included the data from 523 patients in total. The background of the patients is summarized in S5 Table. The resulting Kaplan-Meier plots of each module are depicted in Fig 4. The Cox proportional hazard model results indicated that the representative expression value of module 22 using top-5 kME values was significantly associated with the overall survival (HR = 2.06, 95% CI = 1.46–2.91, p < 0.001), while module 4 and 17 were not significantly associated (HR = 1.15, 95% CI = 0.84–1.57, p = 0.38 and HR = 1.25, 95% CI = 0.89–1.76, p = 0.20, respectively). The genes with the top-5 kME value of module 22 were Canopy FGF Signaling Regulator 3, interferon regulatory transcription factor (IRF) 3, SH3KBP1 Binding Protein 1, exportin 6, and PNKP. This signature of module 22 was significantly related to the outcome compared to the 1,000 randomly selected gene signatures of the same length.

## Discussion

In this study, we conducted a consensus WGCNA on multiple datasets relating to KT-related diseases. We observed similar module differences mainly consisting of immune-related pathways in both BKPyVAN and TCMR. These results concur with previous reports indicating various immune response pathways plays important roles in BKPyVAN [39, 40]. However, the same module was also upregulated in AR and TCMR, suggesting that the changes are not BKPyVAN specific. Histologically, morphological overlapping exists between AR and BKPyVAN with the normal staining [41]. Additionally, a recent report using microarray data and machine learning suggested that an immune-related gene set could not discriminate between TCMR and BKPyVAN despite the fact that BKPyVAN specific gene expression like large T-antigen could discriminate between TCMR and BKPyVAN [7]. We validated that these immune responses are similar in BKPyVAN and TCMR.

Further, we revealed specific differences in BKPyVAN, which were related to the pathways including DDR and translation initiation and regulation. BKPyV evades the cellular machinery of hosts, preventing cell death from the stress caused by infection, by controlling multiple signaling pathways to successfully replicate and survive [42, 43]. The potential mechanism of the upregulation of the DDR pathway is to help repair the DNA damage caused by BKPyV infection, as suggested by the previous experiment [44]. These suggested that in addition to the cell culture experiments, renal biopsy samples from clinically diagnosed BKPyVAN had also upregulated DDR-related genes. An *et al*. recently reported a single-cell transcriptome study of BKPyV infected RPTECs [45]. They revealed that ribosomal protein (RP) genes related to translation were downregulated in cells expressing high-level of viral transcripts while they are upregulated in the condition with medium-level of viral transcripts at 2 days post infection. The current study shows some similarities in that the specific module in the current manuscript, which expression is upregulated in BKPyVAN, contains RP genes and related biological pathways.

The result of IHC implies that the DDR pathway is upregulated during the pathogenesis of BKPyVAN. The importance of phosphorylation of H2AX in BKPyVAN not only in the cell culture but also in the clinical sample was confirmed. The DDR-related proteins were also suggested to be important in the other studies [46, 47]. However, we cannot confirm the direct interaction between BKPyV infection and phosphorylation of H2AX within the same cells [44]. In this study, although the number of cases is small, the IHC results indicate that the staining of the DDR marker alone cannot differentiate AR from BKPyVAN clinically using the histological images. One of the possible reasons is that some other studies reported staining of γH2AX in other kidney diseases, and the severity of the disease could affect the result [48].

Several reports suggested that BKPyV infection is related to tumorgenesis of RCC and urothelial carcinoma (UC). RCC occurs in both native kidneys and transplant kidneys after KT [10, 49]. One study reported the presence of BKPyV DNA in RCC samples [50], and some case reports showed SV40-positive RCC and collecting duct carcinoma [51, 52]. Some mechanisms are proposed for the tumorgenesis by BKPyV. Recent reports indicate that BKPyV infection is a risk factor for UC through the mechanism of integration and APOBEC3-induced genomic instability [53]. While we could not assess how gene expression contributes to the tumorgenesis, we assessed prognostic information of transcriptomic signature specific to BKPyV infection.

Among the top-5 hub genes related to the prognosis of RCC, PNKP is related to DDR, especially for base excision repair, IRF3 is related to the antiviral response by inducing the interferon signaling, involving with the other hub gene in the same module shown in Fig 3, DDX41, which is shown to be required for triggering the interferon signaling in cyclic GMP-AMP synthase-stimulator of interferon genes-IRF3 cascade through DNA sensor [54, 55]. Exportin 6 is reported to be associated with some cancers [56]. The RCC has long been studied and recognized to be an immunogenic tumor, suggested by the heavy immune infiltration [8]. These results suggested that the potential biological link between anti-tumor immunologic responses of RCC and antiviral responses of BKPyVAN through DDR and stimulator of interferon genes might exist, suggested by the result that higher expression of transcriptomic signatures of BKPyVAN was related to poorer survival of RCC. Although the causal relationship is unclear, we suspected that the worse the prognosis, the more compensatory these anti-viral and anti-tumor signals are enhanced, but not fully compensated for. Module 4 which contains mostly immune-related pathways did not show associations with the prognosis of RCC.

In summary, the gene cluster involved in the translation process and DDR pathway was specifically upregulated in BKPyVAN in the clinical renal biopsy samples as shown by consensus gene network analysis. Additionally, the identified module might be related to RCC prognosis. These results could potentially explain the immune responses underlying KT-related diseases and RCC.

## Supporting information

S1 Text The description of datasets. (DOCX)

S2 Text Preprocessing of microarray data and the detail of weighted co-expression network analysis. (DOCX)

S1 Table The enriched pathways for the consensus modules. (DOCX)

S2 Table The identified hub genes. (DOCX)

S3 Table The genes related to DNA damage response in the identified module. (DOCX)

S4 Table The demographic and pathological information of the patients for whose specimens were performed immunohistochemistry. (DOCX)

S5 Table The patient background in survival analysis. (DOCX)

S1 Fig The relationship between soft threshold power, scale-free topology fit index, and mean connectivity. (DOCX)

S2 Fig The hierarchical clustering results of the consensus module eigengenes for each dataset. (DOCX)

S3 Fig The proportion of DNA damage response-related genes in the identified modules. (DOCX)

S4 Fig The immunohistochemistry of the patients with BK polyomavirus-associated nephropathy and the other conditions. (DOCX)

S5 Fig The immunohistochemistry of the patient with BK polyomavirus-associated nephropathy. (DOCX)

S6 Fig The results of module preservation analysis. (DOCX)
