# Supplementary material for: Exploring the mechanism of BK polyomavirus-associated nephropathy through consensus gene network approach
Source: PLoS One. 2023 Jun 15;18(6):e0282534. doi: 10.1371/journal.pone.0282534 (PMC10270345; doi:10.1371/journal.pone.0282534)
Supplement: S1 Text — (DOCX) [file pone.0282534.s001.docx]

**Supplementary Text S1. The description of datasets**

Among Table 1 in the main manuscript, the publicly available datasets were downloaded from the NCBI GEO database [1] and organized as follows.

The study depositing GSE72925 investigated the gene expression signature of BK polyomavirus associated nephropathy (BKPyVAN) compared to the other conditions [2]. In this study, BKPyVAN was diagnosed as polyomavirus PCR positivity in peripheral blood, and simian virus 40 (SV40) staining in the renal allograft biopsy. The other diagnosis criteria were described in the original paper. We selected all the samples including “interstitial fibrosis and tubular atrophy (IFTA)”, “T-cell mediated rejection”, “BKPyV viremia in blood only and no evidence of nephropathy on biopsy (BKPyVB)”, “Normal”, and “BKV nephropathy (BKVN)” from the dataset. The “BKVN” label was renamed to “BKPyVAN” in the analysis.

The study performed by the same group, depositing GSE75693 investigated the relationship between urine proteomics and gene expression profile using microarray in kidney transplantation recipients [3]. We selected all the samples in the dataset for our analysis. Each observation in the data was originally labeled as “Acute rejection (AR)”, “BKVN”, “chronic allograft nephropathy (CAN)”, “no-CAN”, and “Standard (STA)”. We recategorized those with “AR”, “BKPyVAN”, “CAN”, and “Normal” which included the original “no-CAN” and “STA” categories. The diagnostic criteria are described in the original paper. Briefly, the BKPyVAN was diagnosed as the histological confirmation of SV40 staining with or without allograft dysfunction. The platform employed for GSE72925 and GSE75693 was Affymetrix Human Genome U133 Plus 2.0 Array.

The study depositing GSE47199 specifically investigated patients with BKPyVAN and normal transplant kidney patients with (BKPyVB) and without BKPyV viremia [4]. The Affymetrix Human Gene 1.0 ST Array platform was utilized. The diagnostic criteria for these conditions are also described in the original paper. In the latter study, BKPyVAN was diagnosed based on characteristic viral inclusion and positive SV40 immunostaining.

The RNA-seq dataset, GSE120495 was deposited by Wang *et al*. in the study investigating house keeping genes in the kidney-transplant related diseases [5]. The dataset contains 5 samples of standard, acute tubular injury, T-cell mediated rejection, IFTA, BKPyVAN, and interstitial nephritis. We included DESeq-normalized count data of all the samples.

The control category was “Normal” for GSE72925, “Normal” for GSE75693, standard control without BKPyV viremia for GSE47199, and recipients with stable allograft function for GSE120495.

**References**

1. Edgar R, Domrachev M, Lash AE. Gene Expression Omnibus: NCBI gene expression and hybridization array data repository. Nucleic Acids Res. 2002;30: 207–210.

2. Sigdel TK, Bestard O, Salomonis N, Hsieh S-C, Torras J, Naesens M, et al. Intragraft Antiviral-Specific Gene Expression as a Distinctive Transcriptional Signature for Studies in Polyomavirus-Associated Nephropathy. Transplantation. 2016;100: 2062–2070.

3. Sigdel TK, Gao Y, He J, Wang A, Nicora CD, Fillmore TL, et al. Mining the human urine proteome for monitoring renal transplant injury. Kidney Int. 2016;89: 1244–1252.

4. Lubetzky M, Bao Y, O Broin P, Marfo K, Ajaimy M, Aljanabi A, et al. Genomics of BK viremia in kidney transplant recipients. Transplantation. 2014;97: 451–456.

5. Wang Z, Lyu Z, Pan L, Zeng G, Randhawa P. Defining housekeeping genes suitable for RNA-seq analysis of the human allograft kidney biopsy tissue. BMC Med Genomics. 2019;12: 86.
