# Supplementary material for: Exploring the mechanism of BK polyomavirus-associated nephropathy through consensus gene network approach
Source: PLoS One. 2023 Jun 15;18(6):e0282534. doi: 10.1371/journal.pone.0282534 (PMC10270345; doi:10.1371/journal.pone.0282534)
Supplement: S2 Text — (DOCX) [file pone.0282534.s002.docx]

**Supplementary Text S2. Preprocessing of microarray data and the detail of weighted co-expression network analysis**

Raw CEL files for each dataset were downloaded from the NCBI GEO database. CEL files were loaded into R with affy or oligo library. We checked whether there are overlapping samples within and between the datasets by doppelgangeR, and excluded the samples highly similar to the other samples. Eleven sample pairs with highly similar expression patterns between GSE72925 and GSE75693, as well as within GSE72925 were detected, and one of the samples of each pair was excluded beforehand. Additionally, three and two outliers in GSE72925 and GSE75693 were removed from the analysis beforehand. We computed robust multichip average expression measures by function *rma*. We excluded genes with a median intensity of below 3 in more than or equal to 90% of the total sample of each dataset. The principal component analysis was performed to visualize the relationship between conditions. Probe identifiers were annotated using hgu133plus2.db or hugene10sttranscriptcluster.db library. Prior to this, probes that either matching multiple or no gene symbols had been excluded. Subsequently, using the function *goodSapmleGenes* implemented in WGCNA, genes that had multiple missing entries and genes with zero variance were removed. The hierarchical clustering was done using filtered expression values as an input criterion; any height values above 350 were deemed outliers and excluded from further analysis. As the datasets used different platforms, all probes matched to gene symbols were subsequently processed by *collapseRows* function in WGCNA to include only intersected gene symbols in both datasets in subsequent analysis.

The WGCNA first calculates the adjacency matrix by choosing the soft thresholding power to ensure the approximate scale-free topology, and calculate the topological overlap matrix (TOM) to minimize the noises and spurious correlation. Preprocessed and filtered data were used as input, and we first determined soft-thresholding power for all of the datasets. Powers were tested from one to 30 and the scale-free topology fit index (SFTI) and the mean connectivity were calculated. We chose a power that SFTI initially exceeded 0.8 in all datasets. The determined power was used to calculate the TOM. The TOM type “*signed*” was selected, and the type of network was “*signed hybrid*” and the correlation type was the biweight midcorrelation. Subsequently, scaling of TOM was performed, and the consensus TOM was defined by taking the component-wise minimum in each dataset. We subtracted the calculated consensus TOM from one, and the hierarchical clustering was performed. The resulting dendrogram was branch pruned by *cutreeDynamic* function with the parameters of a minimum cluster size of 35 and *deepSplit* of 2. The eigengenes, which corresponded to the first principal component of the expression values of each cluster were calculated, and the correlation matrix of eigengenes was calculated. The matrix was subtracted from one, and again the hierarchical clustering was performed to merge closely related modules with the parameter of the maximum dissimilarity of 0.5.
