# Supplementary material for: Exploring the mechanism of BK polyomavirus-associated nephropathy through consensus gene network approach
Source: PLoS One. 2023 Jun 15;18(6):e0282534. doi: 10.1371/journal.pone.0282534 (PMC10270345; doi:10.1371/journal.pone.0282534)
Supplement: S1 Table — (DOCX) [file pone.0282534.s003.docx]

**Supplementary Table S1. The enriched pathways for the consensus modules**

| **ME** | **ID** | **Description** | **Count** | **pvalue** | **Adjusted pvalue** |
| --- | --- | --- | --- | --- | --- |
| 4 | R-HSA-198933 | Immunoregulatory interactions between a Lymphoid and a non-Lymphoid cell | 51 | <0.001 | <0.001 |
| 4 | R-HSA-449147 | Signaling by Interleukins | 66 | <0.001 | <0.001 |
| 4 | R-HSA-380108 | Chemokine receptors bind chemokines | 24 | <0.001 | <0.001 |
| 4 | R-HSA-6783783 | Interleukin-10 signaling | 20 | <0.001 | <0.001 |
| 4 | R-HSA-6798695 | Neutrophil degranulation | 56 | <0.001 | <0.001 |
| 4 | R-HSA-202430 | Translocation of ZAP-70 to Immunological synapse | 12 | <0.001 | <0.001 |
| 4 | R-HSA-202433 | Generation of second messenger molecules | 15 | <0.001 | <0.001 |
| 4 | R-HSA-877300 | Interferon gamma signaling | 23 | <0.001 | <0.001 |
| 4 | R-HSA-202427 | Phosphorylation of CD3 and TCR zeta chains | 12 | <0.001 | <0.001 |
| 4 | R-HSA-373076 | Class A/1 (Rhodopsin-like receptors) | 43 | <0.001 | <0.001 |
| 4 | R-HSA-913531 | Interferon Signaling | 31 | <0.001 | <0.001 |
| 4 | R-HSA-418594 | G alpha (i) signalling events | 40 | <0.001 | <0.001 |
| 4 | R-HSA-388841 | Costimulation by the CD28 family | 18 | <0.001 | <0.001 |
| 4 | R-HSA-389948 | PD-1 signaling | 11 | <0.001 | <0.001 |
| 4 | R-HSA-375276 | Peptide ligand-binding receptors | 29 | <0.001 | <0.001 |
| 4 | R-HSA-202403 | TCR signaling | 22 | <0.001 | <0.001 |
| 4 | R-HSA-500792 | GPCR ligand binding | 47 | <0.001 | <0.001 |
| 4 | R-HSA-202733 | Cell surface interactions at the vascular wall | 22 | <0.001 | <0.001 |
| 4 | R-HSA-909733 | Interferon alpha/beta signaling | 15 | <0.001 | <0.001 |
| 4 | R-HSA-6785807 | Interleukin-4 and Interleukin-13 signaling | 18 | <0.001 | <0.001 |
| 4 | R-HSA-451927 | Interleukin-2 family signaling | 11 | <0.001 | <0.001 |
| 4 | R-HSA-164952 | The role of Nef in HIV-1 replication and disease pathogenesis | 9 | <0.001 | <0.001 |
| 4 | R-HSA-9658195 | Leishmania infection | 27 | <0.001 | 0.002 |
| 4 | R-HSA-168898 | Toll-like Receptor Cascades | 21 | <0.001 | 0.002 |
| 4 | R-HSA-202424 | Downstream TCR signaling | 15 | <0.001 | 0.005 |
| 4 | R-HSA-1236975 | Antigen processing-Cross presentation | 15 | <0.001 | 0.012 |
| 4 | R-HSA-9664407 | Parasite infection | 11 | <0.001 | 0.013 |
| 4 | R-HSA-9664417 | Leishmania phagocytosis | 11 | <0.001 | 0.013 |
| 4 | R-HSA-9664422 | FCGR3A-mediated phagocytosis | 11 | <0.001 | 0.013 |
| 4 | R-HSA-983695 | Antigen activates B Cell Receptor (BCR) leading to generation of second messengers | 8 | <0.001 | 0.021 |
| 4 | R-HSA-9013149 | RAC1 GTPase cycle | 20 | <0.001 | 0.034 |
| 4 | R-HSA-9660826 | Purinergic signaling in leishmaniasis infection | 7 | <0.001 | 0.04 |
| 4 | R-HSA-9664424 | Cell recruitment (pro-inflammatory response) | 7 | <0.001 | 0.04 |
| 4 | R-HSA-114604 | GPVI-mediated activation cascade | 8 | <0.001 | 0.042 |
| 4 | R-HSA-389359 | CD28 dependent Vav1 pathway | 5 | <0.001 | 0.048 |
| 22 | R-HSA-8868773 | rRNA processing in the nucleus and cytosol | 76 | <0.001 | <0.001 |
| 22 | R-HSA-72312 | rRNA processing | 78 | <0.001 | <0.001 |
| 22 | R-HSA-72613 | Eukaryotic Translation Initiation | 57 | <0.001 | <0.001 |
| 22 | R-HSA-72737 | Cap-dependent Translation Initiation | 57 | <0.001 | <0.001 |
| 22 | R-HSA-72689 | Formation of a pool of free 40S subunits | 52 | <0.001 | <0.001 |
| 22 | R-HSA-6791226 | Major pathway of rRNA processing in the nucleolus and cytosol | 72 | <0.001 | <0.001 |
| 22 | R-HSA-156827 | L13a-mediated translational silencing of Ceruloplasmin expression | 54 | <0.001 | <0.001 |
| 22 | R-HSA-156842 | Eukaryotic Translation Elongation | 49 | <0.001 | <0.001 |
| 22 | R-HSA-1799339 | SRP-dependent cotranslational protein targeting to membrane | 54 | <0.001 | <0.001 |
| 22 | R-HSA-156902 | Peptide chain elongation | 47 | <0.001 | <0.001 |
| 22 | R-HSA-927802 | Nonsense-Mediated Decay (NMD) | 54 | <0.001 | <0.001 |
| 22 | R-HSA-975957 | Nonsense Mediated Decay (NMD) enhanced by the Exon Junction Complex (EJC) | 54 | <0.001 | <0.001 |
| 22 | R-HSA-72706 | GTP hydrolysis and joining of the 60S ribosomal subunit | 53 | <0.001 | <0.001 |
| 22 | R-HSA-192823 | Viral mRNA Translation | 46 | <0.001 | <0.001 |
| 22 | R-HSA-2408557 | Selenocysteine synthesis | 47 | <0.001 | <0.001 |
| 22 | R-HSA-72764 | Eukaryotic Translation Termination | 47 | <0.001 | <0.001 |
| 22 | R-HSA-9633012 | Response of EIF2AK4 (GCN2) to amino acid deficiency | 49 | <0.001 | <0.001 |
| 22 | R-HSA-376176 | Signaling by ROBO receptors | 76 | <0.001 | <0.001 |
| 22 | R-HSA-975956 | Nonsense Mediated Decay (NMD) independent of the Exon Junction Complex (EJC) | 47 | <0.001 | <0.001 |
| 22 | R-HSA-9010553 | Regulation of expression of SLITs and ROBOs | 65 | <0.001 | <0.001 |
| 22 | R-HSA-168273 | Influenza Viral RNA Transcription and Replication | 56 | <0.001 | <0.001 |
| 22 | R-HSA-2408522 | Selenoamino acid metabolism | 51 | <0.001 | <0.001 |
| 22 | R-HSA-168255 | Influenza Infection | 59 | <0.001 | <0.001 |
| 22 | R-HSA-72766 | Translation | 83 | <0.001 | <0.001 |
| 22 | R-HSA-9711097 | Cellular response to starvation | 51 | <0.001 | <0.001 |
| 22 | R-HSA-9679506 | SARS-CoV Infections | 88 | <0.001 | <0.001 |
| 22 | R-HSA-9694516 | SARS-CoV-2 Infection | 71 | <0.001 | <0.001 |
| 22 | R-HSA-72695 | Formation of the ternary complex, and subsequently, the 43S complex | 24 | <0.001 | <0.001 |
| 22 | R-HSA-72662 | Activation of the mRNA upon binding of the cap-binding complex and eIFs, and subsequent binding to 43S | 26 | <0.001 | <0.001 |
| 22 | R-HSA-72203 | Processing of Capped Intron-Containing Pre-mRNA | 62 | <0.001 | <0.001 |
| 22 | R-HSA-9705683 | SARS-CoV-2-host interactions | 54 | <0.001 | <0.001 |
| 22 | R-HSA-9754678 | SARS-CoV-2 modulates host translation machinery | 23 | <0.001 | <0.001 |
| 22 | R-HSA-72649 | Translation initiation complex formation | 25 | <0.001 | <0.001 |
| 22 | R-HSA-69306 | DNA Replication | 51 | <0.001 | <0.001 |
| 22 | R-HSA-69239 | Synthesis of DNA | 38 | <0.001 | <0.001 |
| 22 | R-HSA-72702 | Ribosomal scanning and start codon recognition | 24 | <0.001 | <0.001 |
| 22 | R-HSA-72163 | mRNA Splicing - Major Pathway | 48 | <0.001 | <0.001 |
| 22 | R-HSA-69242 | S Phase | 44 | <0.001 | <0.001 |
| 22 | R-HSA-73884 | Base Excision Repair | 30 | <0.001 | <0.001 |
| 22 | R-HSA-72172 | mRNA Splicing | 48 | <0.001 | <0.001 |
| 22 | R-HSA-69002 | DNA Replication Pre-Initiation | 42 | <0.001 | <0.001 |
| 22 | R-HSA-5357801 | Programmed Cell Death | 50 | <0.001 | <0.001 |
| 22 | R-HSA-68867 | Assembly of the pre-replicative complex | 38 | <0.001 | <0.001 |
| 22 | R-HSA-73894 | DNA Repair | 69 | <0.001 | <0.001 |
| 22 | R-HSA-69190 | DNA strand elongation | 15 | <0.001 | <0.001 |
| 22 | R-HSA-69620 | Cell Cycle Checkpoints | 62 | <0.001 | <0.001 |
| 22 | R-HSA-69206 | G1/S Transition | 35 | <0.001 | <0.001 |
| 22 | R-HSA-1834949 | Cytosolic sensors of pathogen-associated DNA | 22 | <0.001 | 0.001 |
| 22 | R-HSA-453279 | Mitotic G1 phase and G1/S transition | 37 | <0.001 | 0.003 |
| 22 | R-HSA-109581 | Apoptosis | 42 | <0.001 | 0.004 |
| 22 | R-HSA-71291 | Metabolism of amino acids and derivatives | 72 | <0.001 | 0.004 |
| 22 | R-HSA-68886 | M Phase | 78 | <0.001 | 0.005 |
| 22 | R-HSA-5651801 | PCNA-Dependent Long Patch Base Excision Repair | 11 | <0.001 | 0.006 |
| 22 | R-HSA-69052 | Switching of origins to a post-replicative state | 26 | <0.001 | 0.008 |
| 22 | R-HSA-6790901 | rRNA modification in the nucleus and cytosol | 20 | <0.001 | 0.009 |
| 22 | R-HSA-69481 | G2/M Checkpoints | 39 | <0.001 | 0.009 |
| 22 | R-HSA-9020702 | Interleukin-1 signaling | 30 | <0.001 | 0.01 |
| 22 | R-HSA-5693532 | DNA Double-Strand Break Repair | 39 | <0.001 | 0.01 |
| 22 | R-HSA-5357956 | TNFR1-induced NFkappaB signaling pathway | 13 | <0.001 | 0.012 |
| 22 | R-HSA-110373 | Resolution of AP sites via the multiple-nucleotide patch replacement pathway | 12 | <0.001 | 0.012 |
| 22 | R-HSA-73933 | Resolution of Abasic Sites (AP sites) | 15 | <0.001 | 0.014 |
| 22 | R-HSA-72202 | Transport of Mature Transcript to Cytoplasm | 24 | <0.001 | 0.015 |
| 22 | R-HSA-446652 | Interleukin-1 family signaling | 36 | <0.001 | 0.016 |
| 22 | R-HSA-6798695 | Neutrophil degranulation | 85 | <0.001 | 0.019 |
| 22 | R-HSA-157579 | Telomere Maintenance | 29 | <0.001 | 0.02 |
| 22 | R-HSA-193639 | p75NTR signals via NF-kB | 9 | <0.001 | 0.022 |
| 22 | R-HSA-159236 | Transport of Mature mRNA derived from an Intron-Containing Transcript | 22 | <0.001 | 0.022 |
| 22 | R-HSA-162906 | HIV Infection | 48 | <0.001 | 0.023 |
| 22 | R-HSA-5693538 | Homology Directed Repair | 33 | <0.001 | 0.026 |
| 22 | R-HSA-5688426 | Deubiquitination | 58 | <0.001 | 0.026 |
| 22 | R-HSA-379716 | Cytosolic tRNA aminoacylation | 11 | <0.001 | 0.031 |
| 22 | R-HSA-1810476 | RIP-mediated NFkB activation via ZBP1 | 9 | <0.001 | 0.041 |
| 22 | R-HSA-2559583 | Cellular Senescence | 42 | <0.001 | 0.041 |
| 22 | R-HSA-73886 | Chromosome Maintenance | 33 | <0.001 | 0.041 |
| 22 | R-HSA-1606322 | ZBP1(DAI) mediated induction of type I IFNs | 10 | <0.001 | 0.048 |
| 22 | R-HSA-5696397 | Gap-filling DNA repair synthesis and ligation in GG-NER | 11 | <0.001 | 0.049 |
| 17 | R-HSA-196854 | Metabolism of vitamins and cofactors | 27 | <0.001 | <0.001 |
| 17 | R-HSA-196849 | Metabolism of water-soluble vitamins and cofactors | 20 | <0.001 | <0.001 |
| 17 | R-HSA-70895 | Branched-chain amino acid catabolism | 8 | <0.001 | 0.001 |

Count, the number of genes in the module which belong to the pathway; Corrected p-value, p-values corrected by Bonferroni procedure.
