# Supplementary material for: Exploring the mechanism of BK polyomavirus-associated nephropathy through consensus gene network approach
Source: PLoS One. 2023 Jun 15;18(6):e0282534. doi: 10.1371/journal.pone.0282534 (PMC10270345; doi:10.1371/journal.pone.0282534)
Supplement: S2 Table — (DOCX) [file pone.0282534.s004.docx]

**Supplementary Table S2. The identified hub genes**

| **Gene name** | **kME** | **ME** |
| --- | --- | --- |
| LCP2 | 0.896148 | 4 |
| RAC2 | 0.880944 | 4 |
| PARVG | 0.873525 | 4 |
| IKZF1 | 0.856944 | 4 |
| LAIR1 | 0.85403 | 4 |
| ITGB2 | 0.841711 | 4 |
| SP140 | 0.841592 | 4 |
| ARHGAP25 | 0.835097 | 4 |
| CD52 | 0.830226 | 4 |
| PIK3CG | 0.817804 | 4 |
| HCLS1 | 0.81527 | 4 |
| BTK | 0.811279 | 4 |
| JAML | 0.804399 | 4 |
| CYTH4 | 0.79182 | 4 |
| VAV1 | 0.788206 | 4 |
| SLAMF8 | 0.783461 | 4 |
| ITK | 0.782521 | 4 |
| SELL | 0.782448 | 4 |
| HCK | 0.78006 | 4 |
| TRAF3IP3 | 0.777768 | 4 |
| MAP4K1 | 0.77328 | 4 |
| TNFRSF1B | 0.770464 | 4 |
| SELPLG | 0.767696 | 4 |
| SLAMF7 | 0.765868 | 4 |
| GPR18 | 0.763855 | 4 |
| GPR171 | 0.762751 | 4 |
| NLRC5 | 0.761083 | 4 |
| ACOX1 | 0.891 | 17 |
| IQGAP2 | 0.858964 | 17 |
| PCYOX1 | 0.858668 | 17 |
| MCCC2 | 0.823875 | 17 |
| PDE10A | 0.82022 | 17 |
| GRB10 | 0.81878 | 17 |
| HSPA9 | 0.81511 | 17 |
| INSR | 0.81215 | 17 |
| TUB | 0.806334 | 17 |
| WDFY3 | 0.806294 | 17 |
| PDZD8 | 0.802671 | 17 |
| ATP7B | 0.800698 | 17 |
| MAP7 | 0.798555 | 17 |
| ZDHHC9 | 0.797677 | 17 |
| USP51 | 0.794697 | 17 |
| BEND7 | 0.793222 | 17 |
| SFXN1 | 0.792721 | 17 |
| PDZD2 | 0.789816 | 17 |
| TTC39B | 0.788038 | 17 |
| SLC22A5 | 0.787555 | 17 |
| TNFRSF21 | 0.782769 | 17 |
| FRMD3 | 0.781763 | 17 |
| SLC25A44 | 0.778735 | 17 |
| LETM1 | 0.778101 | 17 |
| GPAT3 | 0.776875 | 17 |
| CACHD1 | 0.772253 | 17 |
| MRO | 0.772118 | 17 |
| NET1 | 0.768534 | 17 |
| SLIT2 | 0.765439 | 17 |
| DNAJC6 | 0.764457 | 17 |
| AHCYL2 | 0.764064 | 17 |
| FNIP2 | 0.763138 | 17 |
| MTCH2 | 0.762714 | 17 |
| ILDR2 | 0.762047 | 17 |
| ZNF697 | 0.759931 | 17 |
| EIF4EBP2 | 0.755204 | 17 |
| MAST4 | 0.754464 | 17 |
| DIP2C | 0.754193 | 17 |
| KL | 0.752896 | 17 |
| CNPY3 | 0.881515 | 22 |
| IRF3 | 0.871107 | 22 |
| SHKBP1 | 0.867177 | 22 |
| XPO6 | 0.861958 | 22 |
| PNKP | 0.856701 | 22 |
| PKN1 | 0.855674 | 22 |
| BRPF1 | 0.851247 | 22 |
| NOP2 | 0.847554 | 22 |
| ACD | 0.840342 | 22 |
| NSUN5 | 0.835245 | 22 |
| PGS1 | 0.831492 | 22 |
| MCM7 | 0.830598 | 22 |
| DAGLB | 0.830139 | 22 |
| CCDC97 | 0.828303 | 22 |
| EIF2B1 | 0.826124 | 22 |
| MARS1 | 0.826098 | 22 |
| PIGT | 0.825495 | 22 |
| TRMT1 | 0.82383 | 22 |
| SNRPA | 0.822753 | 22 |
| ARID5A | 0.815691 | 22 |
| ICAM2 | 0.815616 | 22 |
| DDX41 | 0.813618 | 22 |
| CYB561A3 | 0.81343 | 22 |
| RALGDS | 0.811405 | 22 |
| EIF3G | 0.809033 | 22 |
| SMARCAL1 | 0.808812 | 22 |
| TRIM28 | 0.805353 | 22 |
| BYSL | 0.805252 | 22 |
| FHOD1 | 0.805164 | 22 |
| FLII | 0.804932 | 22 |
| YARS1 | 0.804873 | 22 |
| ADRM1 | 0.804036 | 22 |
| KXD1 | 0.803685 | 22 |
| MBOAT7 | 0.802178 | 22 |
| MAP1S | 0.799834 | 22 |
| GRAMD1A | 0.795933 | 22 |
| SART1 | 0.791442 | 22 |
| TFPT | 0.791104 | 22 |
| SRM | 0.78933 | 22 |
| EML3 | 0.787881 | 22 |
| SF3A3 | 0.787709 | 22 |
| RNF31 | 0.787 | 22 |
| ITGB7 | 0.779939 | 22 |
| DPP9 | 0.778446 | 22 |
| ZNF79 | 0.77737 | 22 |
| TAPBPL | 0.777061 | 22 |
| PIK3IP1 | 0.775538 | 22 |
| FKBP15 | 0.773996 | 22 |
| PLEKHM2 | 0.772465 | 22 |
| ENOX2 | 0.77117 | 22 |
| KARS1 | 0.770913 | 22 |
| CANT1 | 0.769873 | 22 |
| GLA | 0.768927 | 22 |
| SLC25A19 | 0.767383 | 22 |
| H2AX | 0.767012 | 22 |
| HDAC1 | 0.766117 | 22 |
| SMG9 | 0.765196 | 22 |
| SNRPB | 0.765125 | 22 |
| KIAA0513 | 0.76499 | 22 |
| EHMT2 | 0.764684 | 22 |
| ELOF1 | 0.764194 | 22 |
| PRKCSH | 0.763645 | 22 |
| ATP13A2 | 0.762488 | 22 |
| LIMK2 | 0.761931 | 22 |
| SF3A2 | 0.759983 | 22 |
| CAD | 0.759949 | 22 |
| FAM53C | 0.75912 | 22 |
| THAP11 | 0.758696 | 22 |
| GTF3C5 | 0.756964 | 22 |
| NUP188 | 0.756277 | 22 |
| PPP6R1 | 0.75528 | 22 |
| PAXX | 0.75456 | 22 |
| SYTL3 | 0.753364 | 22 |
| MCM5 | 0.752075 | 22 |
| C1QTNF6 | 0.750656 | 22 |
| EXOSC10 | 0.750335 | 22 |
