# Supplementary material for: Exploring the mechanism of BK polyomavirus-associated nephropathy through consensus gene network approach
Source: PLoS One. 2023 Jun 15;18(6):e0282534. doi: 10.1371/journal.pone.0282534 (PMC10270345; doi:10.1371/journal.pone.0282534)
Supplement: S3 Table — (DOCX) [file pone.0282534.s005.docx]

**Supplementary Table S3. The genes related to DNA damage response in the identified module**

| **Gene symbol** | **Pathway** |
| --- | --- |
| APEX1 | BER/SSBR |
| APEX2 | BER/SSBR |
| BRCC3 | OTHER |
| CHEK1 | FORK QC |
| DONSON | FORK QC |
| EME1 | HR |
| ERCC3 | NER |
| FANCG | FA/ICL repair |
| FANCI | FA/ICL repair |
| FEN1 | BER/SSBR |
| FEN1 | RER |
| H2AX | EJ |
| HMCES | FORK QC |
| LIG1 | BER/SSBR |
| LIG1 | RER |
| MAD2L2 | EJ |
| MUS81 | HR |
| PARP1 | BER/SSBR |
| PNKP | BER/SSBR |
| PNKP | EJ |
| POLB | BER/SSBR |
| POLD1 | RER |
| POLE | RER |
| POLE4 | FORK QC |
| POLH | OTHER |
| RAD23A | NER |
| RNASEH2A | RER |
| RNASEH2B | RER |
| RNF4 | FORK QC |
| SMARCAL1 | FORK QC |
| TIPIN | FORK QC |
| TOPBP1 | FORK QC |
| TP53BP1 | EJ |
| UBE2T | FA/ICL repair |
| XRCC1 | BER/SSBR |
| XRCC4 | EJ |
| XRCC6 | EJ |
