# Supplementary material for: Exploring the mechanism of BK polyomavirus-associated nephropathy through consensus gene network approach
Source: PLoS One. 2023 Jun 15;18(6):e0282534. doi: 10.1371/journal.pone.0282534 (PMC10270345; doi:10.1371/journal.pone.0282534)
Supplement: S4 Table — (DOCX) [file pone.0282534.s006.docx]

**Supplementary Table S4. The demographic and pathological information of the patients for whose specimens were performed immunohistochemistry**

| **Histological diagnosis** | **Age at biopsy** | **Time after RTx** | **Gender** | **t** | **i** | **g** | **v** |
| --- | --- | --- | --- | --- | --- | --- | --- |
| BKPyVAN | 45 | 2.5 years | Female | 3 | 2 | 0 | 0 |
| BKPyVAN with the suspected concurrent AR | 70 | 3 months | Male | 2 | 1 | 1 | 0 |
| AR1  (acute TCMR) | 23 | 3.3 years | Male | 3 | 3 | 0 | 0 |
| AR2  (acute ABMR and suggestive of acute TCMR) | 77 | 1.6 years | Male | 1 | 3 | 0 | 0 |

BKPyVAN, BK polyomavirus nephropathy; AR, acute rejection; RTx, renal transplantation; TCMR, T-cell mediated rejection; ABMR, antibody-mediated rejection; t, Banff quantitative criteria for tubulitis; i, Banff quantitative criteria for inflammation in non-scarred areas of cortex; g, Banff quantitative criteria for glomerulitis; v, Banff quantitative criteria for intimal arteritis.
