# Supplementary material for: Exploring the mechanism of BK polyomavirus-associated nephropathy through consensus gene network approach
Source: PLoS One. 2023 Jun 15;18(6):e0282534. doi: 10.1371/journal.pone.0282534 (PMC10270345; doi:10.1371/journal.pone.0282534)
Supplement: S5 Table — (DOCX) [file pone.0282534.s007.docx]

**Supplementary Table S5. The patient background in survival analysis**

| **Clinical variables** | **n=523** |
| --- | --- |
| Age, mean (standard deviation) | 60.61 (12.14) |
| Gender = Male, No. (%) | 339 (64.8) |
| Pathologic stage, No. (%) | |
| Stage I | 263 (50.3) |
| Stage II | 54 (10.3) |
| Stage III | 122 (23.3) |
| Stage IV | 84 (16.1) |
| Neoplasm histologic grade, No. (%) | |
| G1 | 14 (2.7) |
| G2 | 228 (43.6) |
| G3 | 206 (39.4) |
| G4 | 75 (14.3) |
