# Supplementary material for: Exploring the mechanism of BK polyomavirus-associated nephropathy through consensus gene network approach
Source: PLoS One. 2023 Jun 15;18(6):e0282534. doi: 10.1371/journal.pone.0282534 (PMC10270345; doi:10.1371/journal.pone.0282534)
Supplement: S1 Fig — (DOCX) [file pone.0282534.s008.docx]

**Supplementary Figure S1. The relationship between soft threshold power, scale-free topology fit index, and mean connectivity**

**
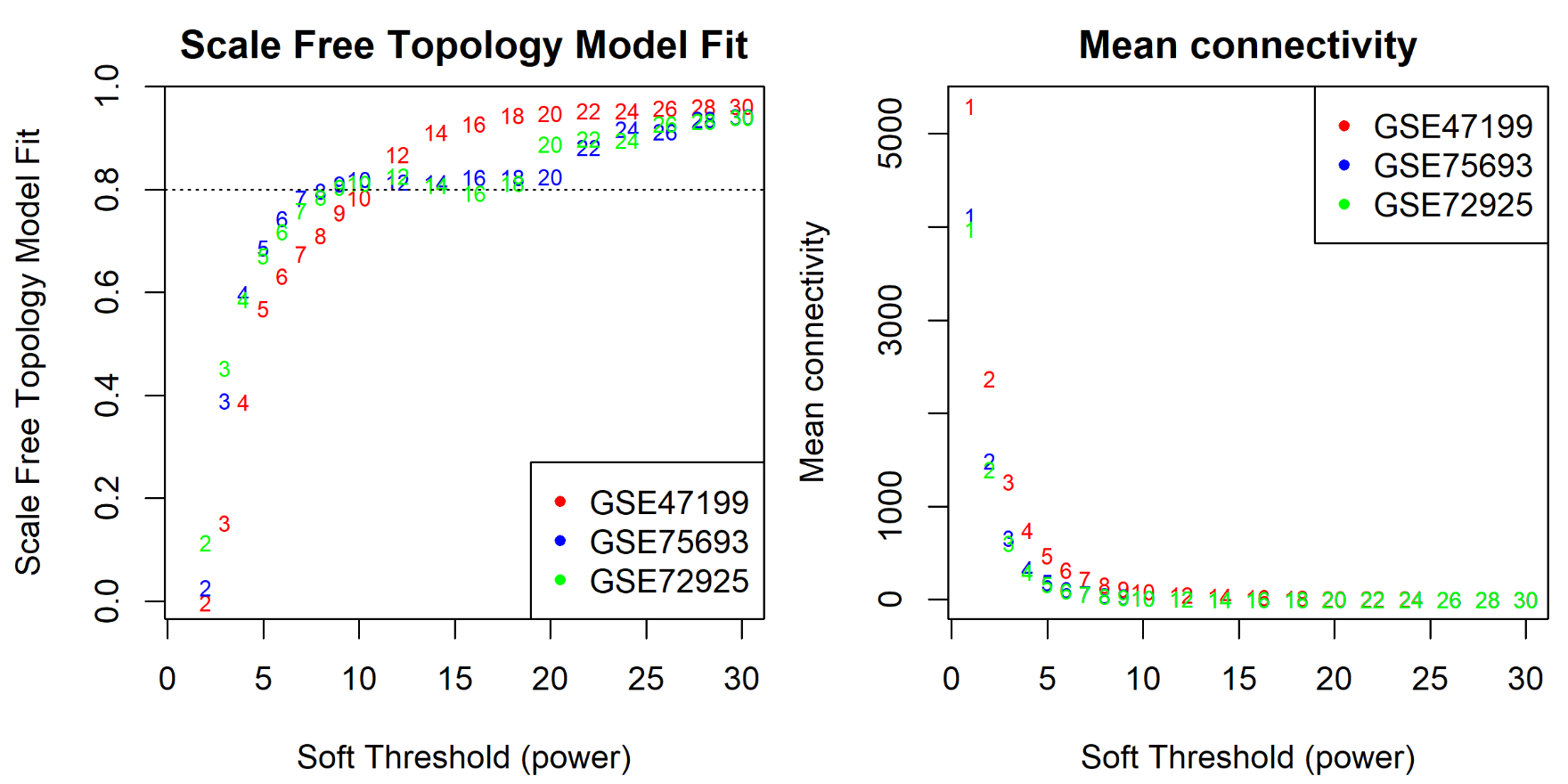
**

The relationship between soft thresholding power, scale-free topology model fit index (SFTI), and mean connectivity for each dataset were visualized. The dotted line indicates SFTI of 0.8.
