# Supplementary material for: Exploring the mechanism of BK polyomavirus-associated nephropathy through consensus gene network approach
Source: PLoS One. 2023 Jun 15;18(6):e0282534. doi: 10.1371/journal.pone.0282534 (PMC10270345; doi:10.1371/journal.pone.0282534)
Supplement: S2 Fig — (DOCX) [file pone.0282534.s009.docx]

**Supplementary Figure S2. The hierarchical clustering results of the consensus module eigengenes for each dataset**
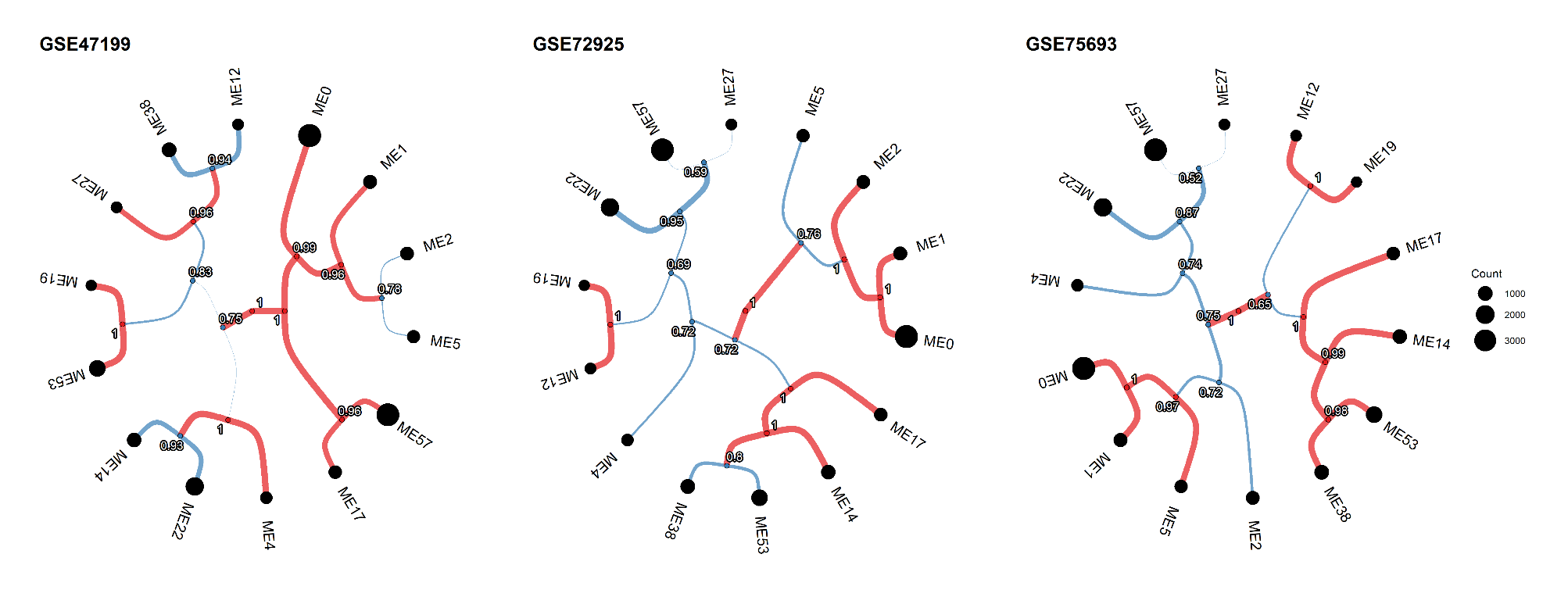


The calculated module eigengenes (ME) are hierarchically clustered by pvclust, and the relationship between the ME is visualized for each dataset. The edge color and the node text represents the significance using the threshold of 0.95 for the approximately unbiased p-value calculated by pvclust. The node size represents the module size.
