# Supplementary material for: Exploring the mechanism of BK polyomavirus-associated nephropathy through consensus gene network approach
Source: PLoS One. 2023 Jun 15;18(6):e0282534. doi: 10.1371/journal.pone.0282534 (PMC10270345; doi:10.1371/journal.pone.0282534)
Supplement: S3 Fig — (DOCX) [file pone.0282534.s010.docx]

**Supplementary Figure S3. The proportion of DNA damage response-related genes in the identified modules
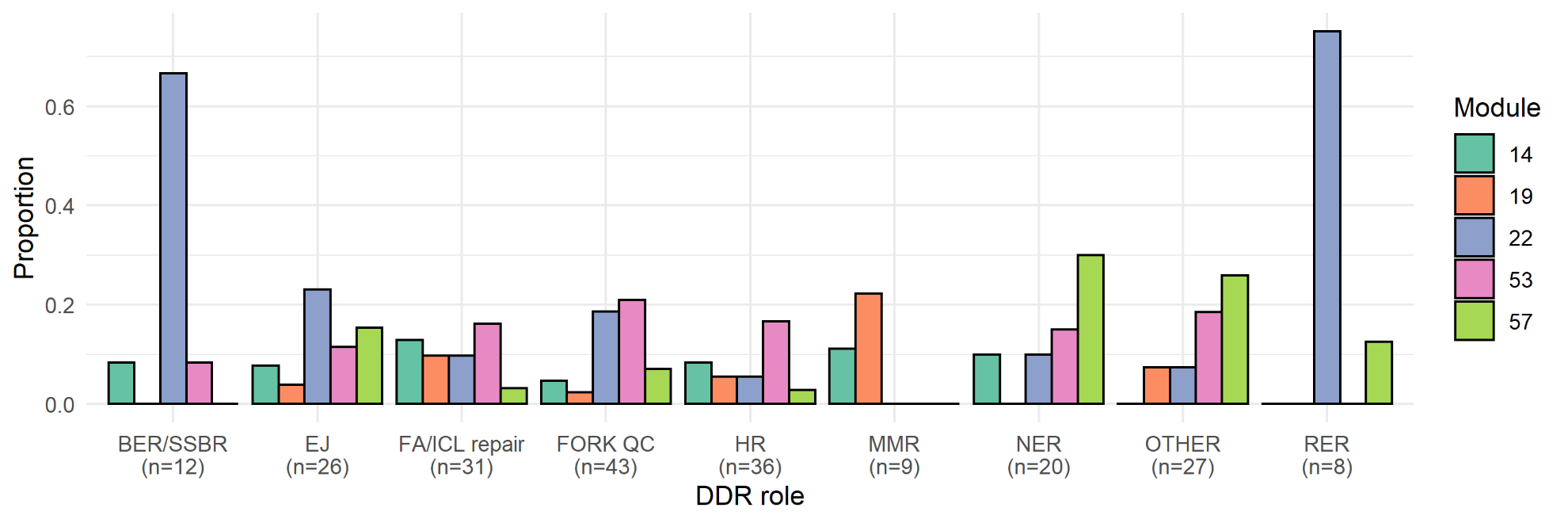
**

The proportions of DNA damage response (DDR) related genes in the modules are shown. Module 0 was discarded beforehand. The modules that have at least 10 DDR-related genes were selected. The category was derived from the paper “A Genetic Map of the Response to DNA Damage in Human Cells”, which is also cited in the main manuscript [1]. BER, base excision repair; DDR, DNA damage response; EJ, end-joining; FA, Fanconi anemia; HR, homologous recombination; ICL, interstrand crosslinks; MMR, mismatch repair; NER, nucleotide excision repair; QC, quality control; RER, ribonucleotide excision repair; SSBR, single-strand break repair.

**Reference**

1. Olivieri M, Cho T, Álvarez-Quilón A, Li K, Schellenberg MJ, Zimmermann M, et al. A Genetic Map of the Response to DNA Damage in Human Cells. Cell. 2020;182: 481-496.e21.
