# Supplementary material for: Exploring the mechanism of BK polyomavirus-associated nephropathy through consensus gene network approach
Source: PLoS One. 2023 Jun 15;18(6):e0282534. doi: 10.1371/journal.pone.0282534 (PMC10270345; doi:10.1371/journal.pone.0282534)
Supplement: S4 Fig — (DOCX) [file pone.0282534.s011.docx]

**Supplementary Figure S4. The immunohistochemistry of the patients with BK polyomavirus-associated nephropathy and the other conditions**


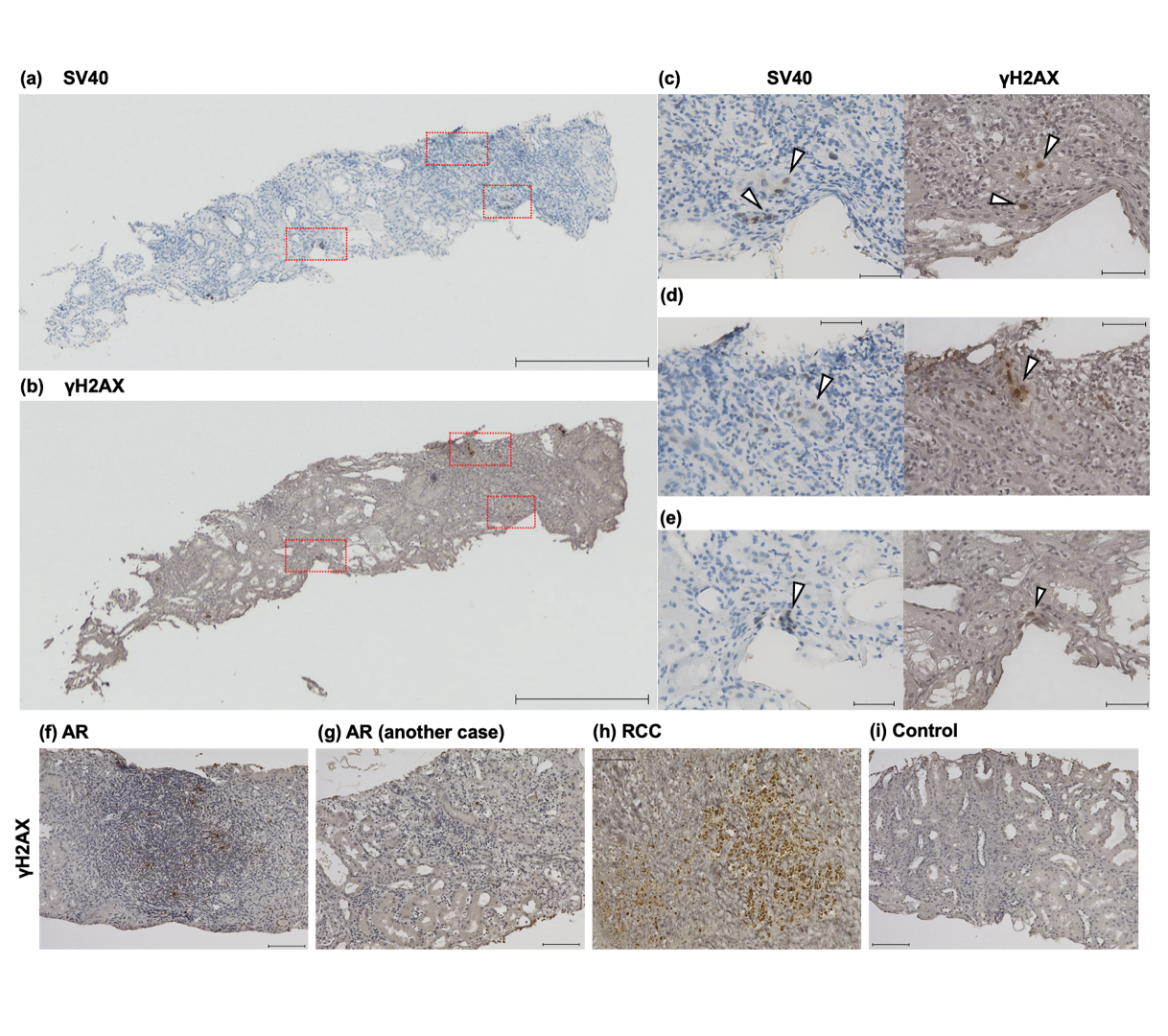


(a), (b) SV40 and γH2AX signals by serial section in the patient with BK polyomavirus associated nephropathy (BKPyVAN). The red dotted square indicates the regions with the signal. (c)―(e) At higher magnification, γH2AX signals are mainly detected in tubules and the nearby cells were positive for SV40 signals (arrowheads). (f)―(i) γH2AX signals in other samples. The specimen of acute rejection (AR) had a strong signal in infiltrating cells. However, in another AR without any evidence of BKPyVAN, only faint γH2AX signals in interstitial areas are observed. The specimen of renal cell carcinoma (RCC) had also a focal strong signal, and the specimen of control (no pathological finding) biopsy only showed faint signals. Scale bar: (a), (b) 500μm; (c)―(e) 50μm; (f)―(i) 100μm. AR, acute rejection; RCC, renal cell carcinoma.
