# Supplementary material for: Exploring the mechanism of BK polyomavirus-associated nephropathy through consensus gene network approach
Source: PLoS One. 2023 Jun 15;18(6):e0282534. doi: 10.1371/journal.pone.0282534 (PMC10270345; doi:10.1371/journal.pone.0282534)
Supplement: S5 Fig — (DOCX) [file pone.0282534.s012.docx]

**Supplementary Figure S5. The immunohistochemistry of the patient with BK polyomavirus-associated nephropathy**


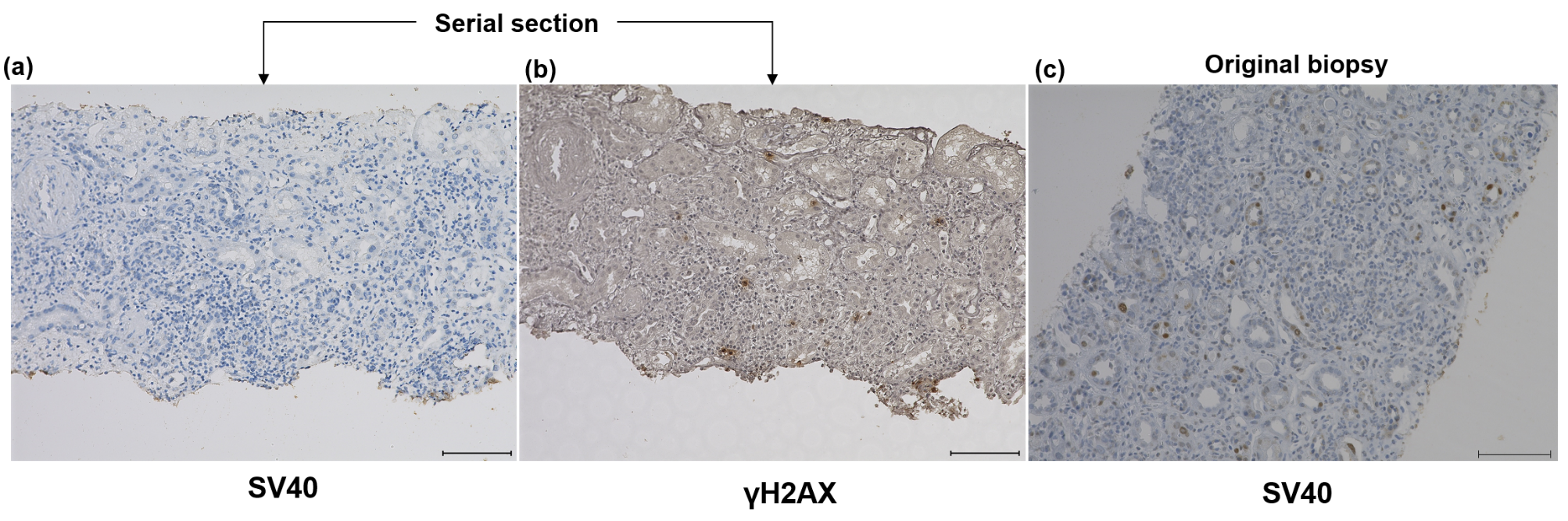


In BK polyomavirus-associated nephropathy with the suspected concurrent acute rejection, no evident SV40 staining was observed whereas tubular γH2AX signals are frequently observed in the serial section cut out in this study. SV40 staining was observed at the time of the original biopsy. Scale bar: 100μm.
