# Supplementary material for: Exploring the mechanism of BK polyomavirus-associated nephropathy through consensus gene network approach
Source: PLoS One. 2023 Jun 15;18(6):e0282534. doi: 10.1371/journal.pone.0282534 (PMC10270345; doi:10.1371/journal.pone.0282534)
Supplement: S6 Fig — (DOCX) [file pone.0282534.s013.docx]

**Supplementary Figure S6. The results of module preservation analysis**


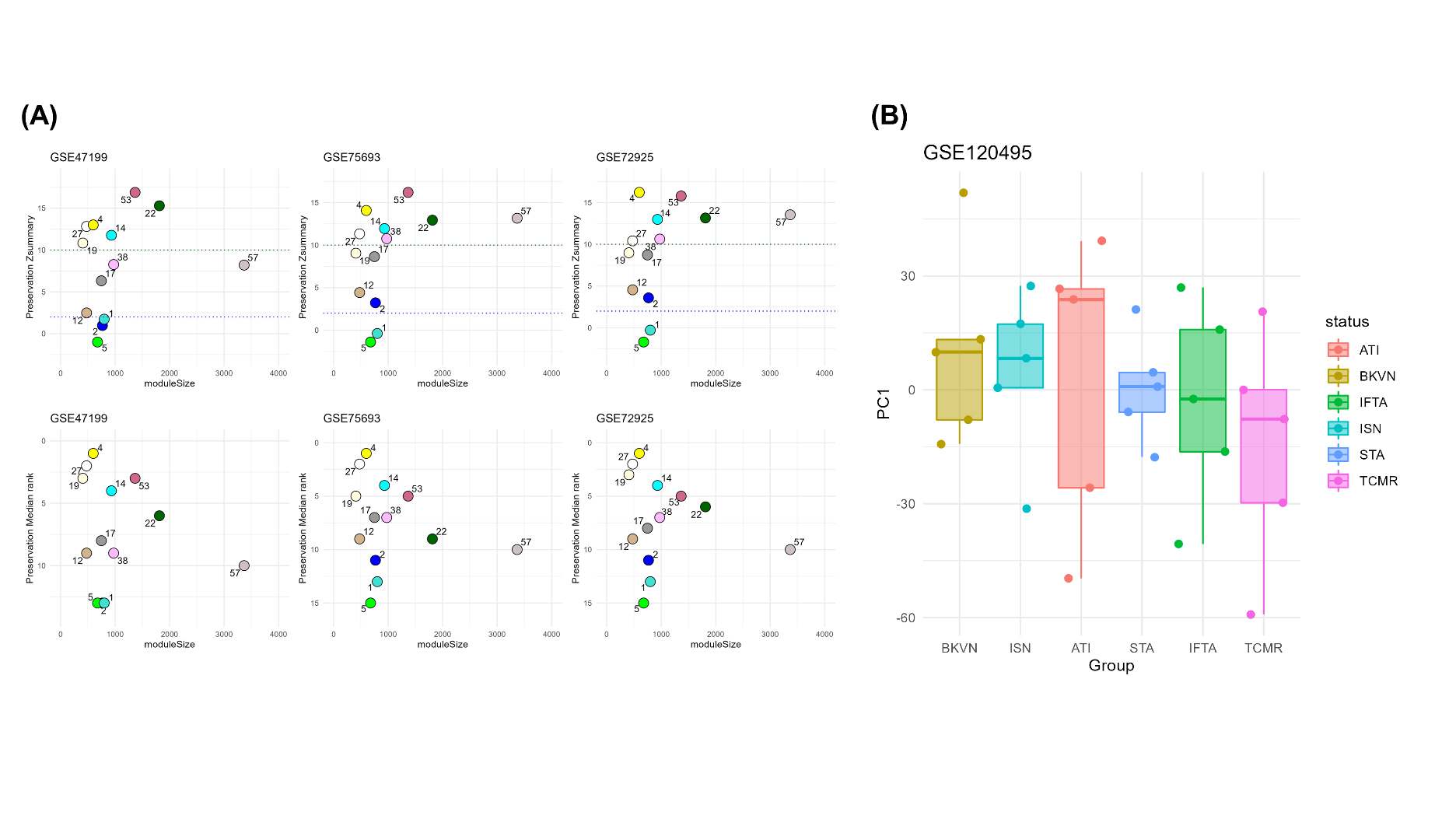


The figure summarizing the module preservation statistics and module eigengene of module 22 in RNA-Seq dataset. (A) The preservation Z-summary and preservation median rank are shown. X-axis represents module size and y-axis shows statistics. (B) The module eigengene of each disease condition is shown, ordered by their mean values.
